# Signet ring cells in carcinomatous lymphangitis due to gastric adenocarcinoma^☆^^☆☆^

**DOI:** 10.1016/j.abd.2019.12.004

**Published:** 2020-05-05

**Authors:** Beatriz da Silva Souza, Renan Rangel Bonamigo, Gabriela Lusa Viapiana, André Cartell

**Affiliations:** a Dermatology Service, Hospital de Clínicas de Porto Alegre, Porto Alegre, RS, Brazil; b Department of Pathology, Faculdade de Medicina, Universidade Federal do Rio Grande do Sul, Porto Alegre, RS, Brazil

**Keywords:** Carcinoma, signet ring cell, Lymphangitis, Lymphatic metastasis

## Abstract

Cutaneous metastases are rare. They usually present as nodules or tumors. Diagnosis is based on histopathological examination and prognosis is unfavorable. This report describes the case of a female patient, 72 years old, with surgically treated gastric antrum adenocarcinoma. Pathology showed poorly differentiated adenocarcinoma with signet ring cells. It evolved with bone involvement, lymph node enlargement in the inguinal region, and skin infiltration in the lower limbs, abdomen, and root of the upper limbs. Skin biopsy demonstrated signet ring carcinoma embolizing the dermal and hypodermic vessels and invasion of adipose tissue, confirming carcinomatous lymphangitis. Carcinomatous lymphangitis is the cutaneous and subcutaneous lymphatic invasion by tumor cells. Cutaneous metastasis is relatively uncommon and presents mainly as cutaneous or subcutaneous nodules, and more rarely as inflammatory lesions. The present case reports carcinomatous lymphangitis associated with gastric cancer.

## Introduction

Lymphedema should be evaluated for different etiologies, including solid organ cutaneous metastases.

Cutaneous metastases are relatively rare dermatological malignancies, with a reported incidence ranging from 0.7% to 9.0% among all cutaneous neoplasms.^1^ They often present as nodules or tumors, mostly erythematous, discrete, and solid; inflammatory skin metastases are infrequent.^2^ The diagnosis is based on histopathological examination.^3^ The prognosis of patients with cutaneous metastasis is unfavorable, with a mean survival of approximately 7.5 months.^3^ This report presents the case of a patient with signet ring cells gastric antrum adenocarcinoma, who developed carcinomatous lymphangitis.

## Case report

**Figure 1:**
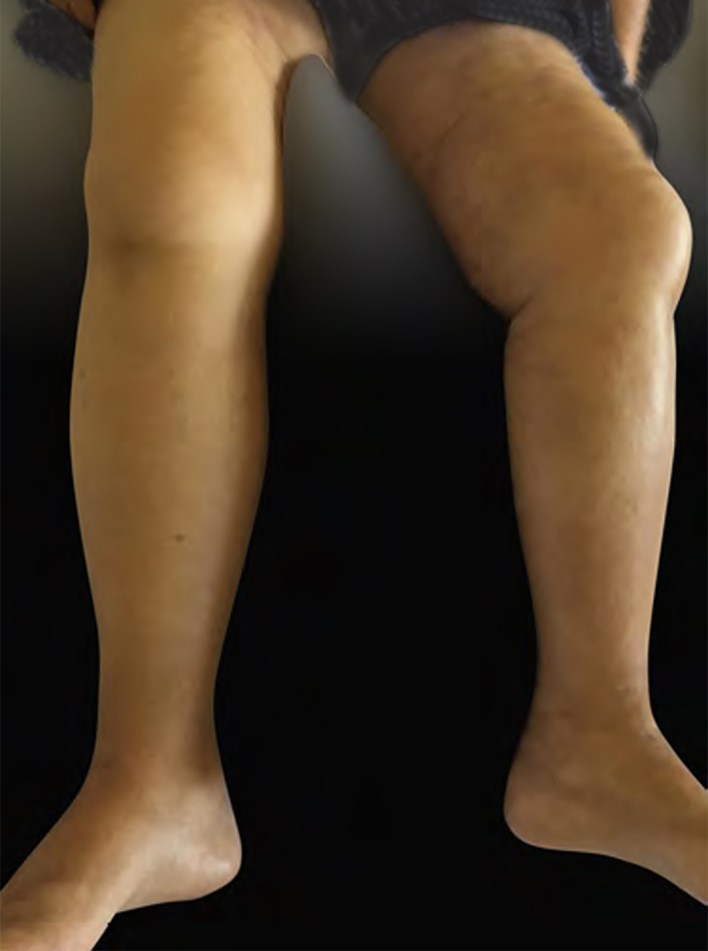
Cutaneous infiltration of the lower limbs, more extensive on the left side, and on the right side affecting the root of the thigh.

**Figure 2:**
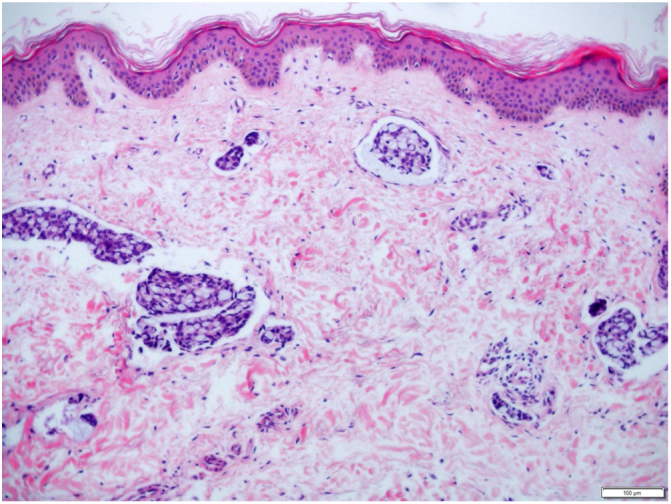
Presence of diffuse lymphatic embolization associated with dermal edema (Hematoxylin & eosin, x50).

**Figure 3:**
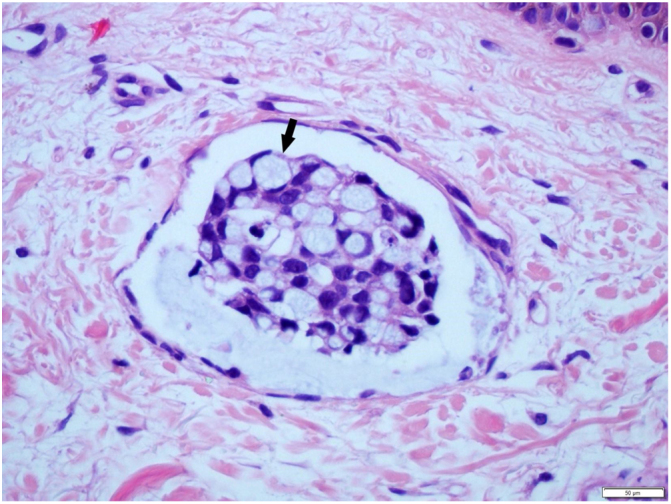
Greater detail showing lymphatic embolus with signet ring cells (indicated by the arrow) (Hematoxylin & eosin, x200).

Female, 72 years old, diagnosed with stage IIIA gastric antrum adenocarcinoma. She underwent partial gastrectomy with enlarged lymphadenectomy, with histopathological evidence showing poorly differentiated adenocarcinoma, mixed type in Laurén's classification, with signet ring cells, ulcero-infiltrative linitis plastica-type, compromising the gastric antrum and pylorus. Adjuvant chemotherapy with capecitabine + oxaliplatin was started, but there was intolerance and therapy was discontinued. A computed tomography (CT) scan performed ten months after surgery showed osteolytic lesion on T2 and lymph node enlargement in the left inguinal region. Palliative radiotherapy was performed for bone lesions, with significant improvement in local pain. After two months, there was volume increase in the left lower limb, which progressed with bilateral involvement; in five months, she was hospitalized due to lower limb lymphedema. On physical examination, she presented cutaneous infiltration in the lower limbs, more pronounced on the left side (Fig. 1), with extension to the abdomen and root of the upper limbs. The histopathological examination of the skin of the medial surface of the left thigh showed signet ring carcinoma embolizing vessels throughout the dermis and hypodermis, with neoplastic invasion of adipose tissue (Figure 2, Figure 3), confirming carcinomatous lymphangitis. Palliative chemotherapy started, but the patient progressed to respiratory failure and eventually death.

## Discussion

Lymphedema is a clinical condition marked by increased volume of subcutaneous soft tissues due to impairment of the lymphatic system.^2^ Lower limb edema is a very common symptom; the mechanisms most often involved in its cause include venous and lymphatic disease, volume overload, increased capillary permeability, and decreased oncotic pressure. The most common associated diseases are deep vein thrombosis and chronic venous insufficiency, heart failure, hepatic or renal failure hypoproteinemia, idiopathic cyclic edema, and drug-induced edema.^4^

In cancer patients, the main etiologies of lymphedema are complications after lymphadenectomy or after radiotherapy. Carcinomatous lymphangitis exhibits extensive cutaneous and subcutaneous lymphatic invasion by tumor cells, caused by lymphogenic dissemination.^2^ When lymphoedema is diagnosed in patients previously treated for malignancy, it is important to consider whether the symptom corresponds to disease progression or recurrence, or a complication of the initial cancer treatment, and histopathology is very important for etiological differentiation. Lymphedema due to malignant infiltration should be considered, particularly when it develops rapidly; it is constantly present, with visible dilated veins and is associated with severe pain.^2^

The signet ring pattern defines a specific cell shape change, during which the cell nucleus is pushed to the periphery due to cytoplasmic accumulation of mucin, vacuoles, or inclusion bodies.^1^ Metastatic skin biopsy of this type of cancer usually demonstrates infiltration of the affected site, in the present case of the dermis and hypodermis, by inflammatory cells and small rounded mononuclear cells with large cytoplasm, occasionally with the appearance of a signet ring.^5^, ^6^

The histological type most prone to distant metastasis is signet ring cell adenocarcinoma.^7^ Perisse et al. reported a case of poorly differentiated antrum adenocarcinoma with signet ring cells with cutaneous metastasis, presenting as asymptomatic nodules on the face, neck, chest, and scrotum.^7^

In general, cutaneous metastases occur in the final course of the disease, but may also be the presenting sign of underlying cancer, related both to poor prognosis and decreased survival.^8^, ^9^ These lesions are often cutaneous or subcutaneous, normochromic or erythematous nodules, often asymptomatic,^2^, ^10^ rarely presenting as inflammatory metastases,^2^ as in the present case, in which cutaneous metastasis manifested as a carcinomatous lymphangitis.

## Financial support

None declared.

## Authors’ contributions

Beatriz da Silva Souza: Drafting and editing of the manuscript; intellectual participation in the propaedeutic and/or therapeutic conduct of the studied cases.

Renan Rangel Bonamigo: Intellectual participation in the propaedeutic and/or therapeutic conduct of the studied cases.

Gabriela Lusa Viapiana: Intellectual participation in the propaedeutic and/or therapeutic conduct of the studied cases.

André Cartell: Intellectual participation in the propaedeutic and/or therapeutic conduct of the studied cases.

## Conflicts of interest

None declared.
